# IRDC-Net: Lightweight Semantic Segmentation Network Based on Monocular Camera for Mobile Robot Navigation

**DOI:** 10.3390/s23156907

**Published:** 2023-08-03

**Authors:** Thai-Viet Dang, Dinh-Manh-Cuong Tran, Phan Xuan Tan

**Affiliations:** 1 Department of Mechatronics, School of Mechanical Engineering, Hanoi University of Science and Technology, Hanoi 10000, Vietnam; cuong.tdm187419@sis.hust.edu.vn; 2 Graduate School of Engineering and Science, Shibaura Institute of Technology, Toyosu, Koto-ku, Tokyo 135-8548, Japan; tanpx@shibaura-it.ac.jp

**Keywords:** computer vision, mobile robot, navigation, obstacle avoidance, semantic segmentation

## Abstract

Computer vision plays a significant role in mobile robot navigation due to the wealth of information extracted from digital images. Mobile robots localize and move to the intended destination based on the captured images. Due to the complexity of the environment, obstacle avoidance still requires a complex sensor system with a high computational efficiency requirement. This study offers a real-time solution to the problem of extracting corridor scenes from a single image using a lightweight semantic segmentation model integrating with the quantization technique to reduce the numerous training parameters and computational costs. The proposed model consists of an FCN as the decoder and MobilenetV2 as the decoder (with multi-scale fusion). This combination allows us to significantly minimize computation time while achieving high precision. Moreover, in this study, we also propose to use the Balance Cross-Entropy loss function to handle diverse datasets, especially those with class imbalances and to integrate a number of techniques, for example, the Adam optimizer and Gaussian filters, to enhance segmentation performance. The results demonstrate that our model can outperform baselines across different datasets. Moreover, when being applied to practical experiments with a real mobile robot, the proposed model’s performance is still consistent, supporting the optimal path planning, allowing the mobile robot to efficiently and effectively avoid the obstacles.

## 1. Introduction

Mobile robots (MRs) safely navigate their environments by recognizing obstacles in real time. MR’s navigation assistance systems detect obstacles through laser scanners [1], sensors [2], and cameras [3]. The navigation systems of complex environments are prohibitively expensive, as they require a considerable amount of computing power [2,3]. The use of Lidar or cameras has become widespread recently. Lidar automatically measures the distance to obstacles, detects the object’s boundary regions, and maintains an MR’s perception of the environment [4]. However, environmental conditions such as lighting, fog, or rain can negatively influence the process of collecting Lidar data.

Furthermore, many kinds of obstacles in indoor environments block or decline laser beams, making the representation of moving environments problematic [5]. To overcome the limitation of Lidar in indoor environments, Lidar can be combined with other sensors or camera systems to improve the data collection ability [4,5,6,7]. Alternatively, cameras offer inexpensive scene data to detect any object [8,9]. Due to the prevalence of affordable, high-precision monocular cameras, previously existing drawbacks have been eliminated. Thus, real-time image segmentation and MR’s path planning have been accomplished [8,10].

Semantic segmentation utilizing deep learning (DL) is a fundamental challenge in many vision-based applications [11,12,13,14,15], including scene interpretation, object detection, and mobile robot perception. Rusli et al. [16] used a Canny edge algorithm, and Houghline transforms to navigate MRs, where the road and obstacles had been detected by vanishing points. Minae et al. [17] investigated DL-based semantic segmentation, demonstrating remarkable performance in experimental segmented images to address the lack of training datasets for evaluating performance. However, the procedure achieved a high processing speed but had relatively low accuracy when environmental factors changed. To enhance the segmentation model’s performance, Shelhamer et al. [18] converted modern classification networks (AlexNet, VGGNet, and GoogLeNet) into fully convolutional networks (FCN). With the same objective, Wang et al. [19,20] introduced a convolutional neural network (CNN) of Adaptive Feature Fusion Unet. However, the use of VGG-FCN models such as [18] or Unet [19] is unsuitable for infrastructure deployment due to their high computational cost.

In this paper, we propose a novel lightweight segmentation model that integrates an FCN decoder and MobilenetV2 encoder for semantic segmentation supporting mobile robot navigation. By leveraging a trained model from antecedent networks and multi-scale fusion in the decoder block, the segmentation process is highly accurate with a low computation time. Specifically, with the use of MobilenetV2, our approach reduces the need for extensive training parameters, from 22 million to 3.3 million in IRDC, in comparison with our previous works [7,10]. Additionally, we enhance performance and computational efficiency via the Adam optimizer. To ensure high-quality input images, we pre-process the dataset with Gaussian blur and Gaussian noise. Importantly, we propose to use the Balanced Cross-Entropy loss function to better handle unbalanced datasets. This results in a more efficient and effective segmentation model that enables the design of the MR’s optimal path planning algorithm, which incorporates local search algorithms to avoid static and dynamic obstacles while tracking global path planning. The experimental results demonstrate that the proposed model can outperform baselines in terms of accuracy and mIoU across three published image datasets. On top of that, the efficacy of the model is maintained when it is tested using a self-collected TaQuangBuu library dataset. Based on this, we could effectively apply perspective correction to construct the MR’s frontal view. In the frontal view, MRs can use the local search algorithms to detect obstacles and track the global path for real-time movement. In practical experiments, it is proved that by using the proposed segmentation model, the smoothness of MR’s trajectory is enhanced while maintaining the minimum changing steering angle in comparison to previous works [7,10].

The main contributions of the proposed approach are as follows:

- Integration of a lightweight FCN decoder (with multi-scale fusion) and MobilenetV2 encoder for efficient segmentation.
- Further enhance performance and computational efficiency by using the Adam optimizer and quantization. In addition, data preprocessing is also improved with the use of appropriate filters.
- Replacement of the Binary Cross Entropy loss function with the Balanced Cross-Entropy loss function for better handling of unbalanced datasets.
- The proposed model is compared with a number of baselines across several datasets, followed by a practical evaluation with a mobile robot.

The paper is organized as follows: Section 2 provides related works. The proposed model is introduced in Section 3. Section 4 presents the experiment and results. Section 5 concludes the paper.

## 2. Related Works

In this section, we present several encoder-decoder architectures, i.e., SegNet, Enet, and U-Net, used in semantic segmentation tasks. These architectures are the main inspiration for us to introduce IRDC-Net for real-time mobile robot navigation. Since these architectures are based on the concept of Fully Convolutional Networks (FCNs), this section will be initiated with the description of FCNs.

### 2.1. Fully Convolutional Networks (FCNs)

CNNs have traditionally been used to classify images by employing convolutional layers for feature extraction and fully connected layers for classification [21]. However, in the image segmentation problem, the goal is to predict the label for each pixel instead of classifying the entire image. One of the well-known segmentation models is the FCN network [22,23], which replaces fully connected layers with 1 × 1 convolutional layers to generate an output with a similar size to the input. As a result, the FCN network can take input images of varying sizes and produce a segmented heatmap for the entire image. Its network, in fact, comprises two main components: the encoder and the decoder. The encoding process uses convolutional layers to extract meaningful image features and reduce the output size. The decoder, on the other hand, uses deconvolutional (transposed convolution) layers and skip connections, to generate a high-resolution, segmented heatmap with a high degree of detail.

### 2.2. SegNet

SegNet [24] is a CNN architecture specifically designed for segmentation. Its architecture is at identifying and classifying regions within an image and assigning labels to each pixel. The coding part of SegNet uses convolutional layers to extract features from an input image. The decoding part, using deconvolution layers, reconstructs the segmented image from the high-level representation obtained from the decoder. One of the distinctive features of SegNet is the use of “skip connections”, which allows information to be retained from the encryption layers and passed to the decryption layers [25]. As the result, the recovery of the final segmented image’s detail can be enhanced.

### 2.3. ENet

ENet (Efficient Neural Network) [26] is another CNN-based segmentation model. It is a lightweight and optimized network for real-time image processing on devices with limited computational resources, such as embedded applications and mobile devices. It has a unique architecture designed to reduce its parameters, increase computation speed, and maintain a high level of segmentation quality. ENet utilizes various techniques, including sparse connections, pointwise connections, and block pooling techniques, to decrease the size of computational graphs, decrease computational burden, and increase training speed and forecasting [27].

### 2.4. U-Net

U-Net [19] is an extensively used CNN architecture for image segmentation. The U-Net network has a unique architecture consisting of an encoder and decoder. The encoding part of U-Net uses convolutional and pooling layers to extract image features and reduce output size. Specifically, U-Net’s architecture includes many successive convolutional layers with decreasing size and an increasing number of channels to generate an image’s high-level feature map. U-Net’s decoding section utilizes deconvolution (transposed convolution) layers to expand the input and reconstruct the segmented image. In addition, U-Net integrates data from multiple encryption layers using skip connections to pass information from encryption to decryption. The combination of the encoding and decoding portions of U-Net contributes to the creation of a unique “U” network architecture, which resembles the shape of the underlying structure (U-shape) and is therefore referred to as U-Net [20].

## 3. Lightweight Semantic Segmentation FCN-MobilenetV2

FCN-MobilenetV2′s architecture and model training comprised most of the lightweight semantic segmentation systems. Section 3.1, Section 3.2 and Section 3.3 describe additional information of network architecture, model training, and quantization for this proposed method.

### 3.1. Network Architecture

**Figure 1:**
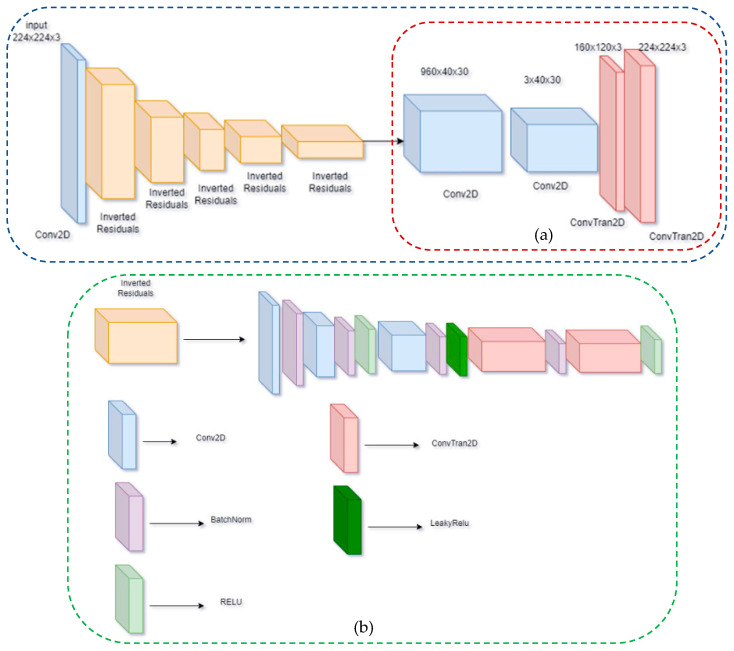
Proposed lightweight semantic segmentation FCN-MobilenetV2 with (**a**) the architecture of Deepwise Convolution and (**b**) the detailed architecture of Inverted Residuals.

The proposed model is based on the MobilenetV2 network [28], as shown in Figure 1. Model input is a form of 224 × 224 images, and 15 inverted residual (IR) blocks are used to extract image features. In comparison to the conventional residual mechanism, the feature extraction process is improved by increasing the number of convolutional layers in the intermediate layers. In addition, the IR blocks are connected to depth-wise conv (DC) classes to reduce the number of model parameters.

**Figure 2:**
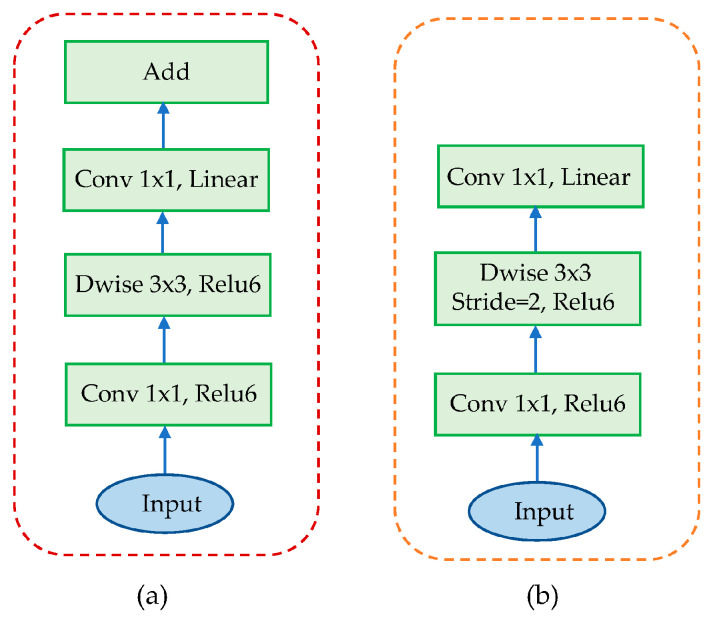
MobilenetV2 architecture with (**a**) Strike = 1 block and (**b**) Strike = 2 block.

**Figure 3:**
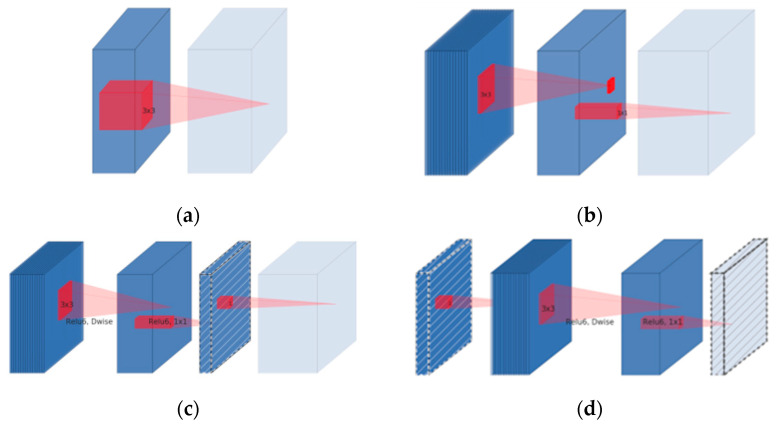
Model of separable convolutional blocks [28] with (**a**) Regular, (**b**) Separable, (**c**) Separable with linear bottleneck, and (**d**) Bottleneck with expansion layer.

The IRDC-Net architecture, which consists of the IR and DC is depicted in Figure 2. The first layer presents a 1 × 1 convolution with Relu6 activation function. The second layer is identical to the following 3 × 3 depthwise convolution as a DC to reduce its parameters. Following this, the third layer is 1 × 1 convolution without any activation function. “Linear” block is replaced by “Relu” Block. The architecture uses two residual blocks with “stride = 1” and “stride = 2” to serve intermediate layers. The difference between IR and the original one lies in the adjustment of the skip connection used in MobilenetV2. The IR requires fewer input and output channels for each residual block (bottleneck layer) [28], as shown in Figure 3.

The IR blocks in IRDC-Net compress linked the layers where the skip connections are connected. In contrast, the original residuals used in ResNet [29,30] have input and output channels that are more than that of the intermediate layers. In Figure 3c,d, linear bottleneck and inverted residual blocks between bottlenecks are also recommended in addition to MobileNetV2 depth-separable structures.

As for DC, instead of using a single kernel (filter) to conduct convolution computations on the entire input channel, it employs a different kernel for each input channel. This allows us to reduce the number of parameters and computations, as we only need to compute the convolution on a single channel at a time rather than on all channels. We can use a standard convolution layer; features from separate channels can be combined.

In the proposed semantic segmentation model, the decoder’s architecture is constructed as follows:

- 1 conv2d layer (1280 × 1000 × 7 × 7): Synthesized feature output from the classifier class of MobileNet.
- 1 class conv2d (1280, num_classes, kernel_size = 1): This layer condenses the model’s features.
- 1 class convTranpos2D (numclass, numclass): Scale output of the model.
- 1 Class ConvTranpos2D: This scales the output of the model. Build a model based on FCN network architecture, help upscaling output equal to input size, and classify each image pixel into separate classes.

### 3.2. Model Training

**Figure 4:**
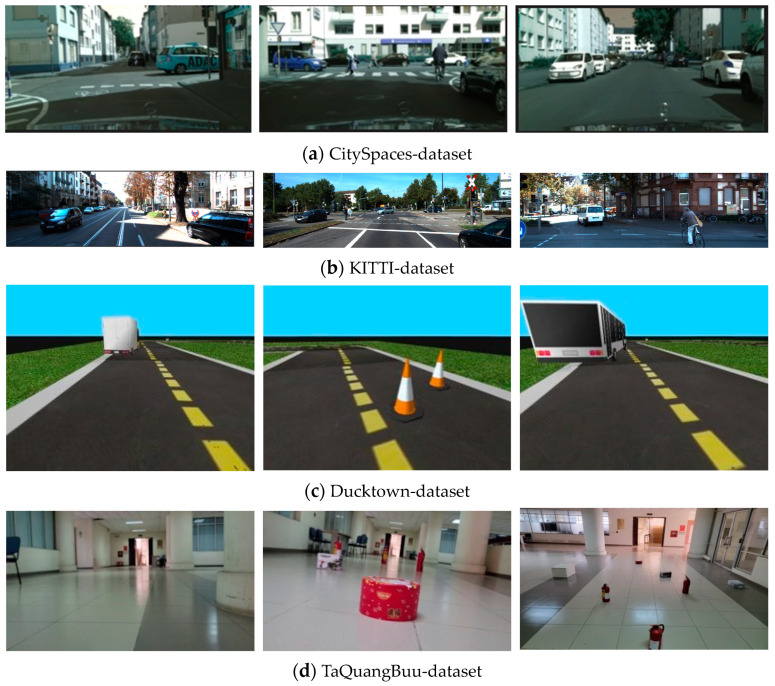
Raw images from four datasets of CitySpaces [31], KITTI [32], Ducktown [33], and TaQuangBuu’s dataset.

The proposed model was trained on four datasets (Figure 4), which include CitySpaces (5000 images) [31], KITTI (400 images) [32], Duckie-dataset collected from Ducktown (1200 images) [33], and TaQuangBuu’s dataset consisting of 1200 images collected from Ta Quang Buu library supplied in the Data Availability Statement.

Our experiments are carried out with the following configuration: Python 3.11.0; TensorFlow 1.4 framework; a computer with Core I7 11th generation processor 2.50 GHz, Nvidia 2080TI graphics card with 12 GB VRAM, 32 GB RAM, and a 64-bit operating system. 

According to the actual data of the TaQuangBuu library image updating the self-collected images in [7,10], the ratio of pixels that should be on the path to pixels that should be obstacles is quite high. In our previous studies [7,10], the Binary Cross Entropy loss function for two classes, such as available and unavailable regions, was used. However, this led to an imbalance in the data. When using the Binary Cross Entropy as loss function, the learning model tends to favor the object that appears more frequently in the data. Adding more instances of the less dominant class to training data might potentially solve the problem. Therefore, we propose to use the Balanced Cross-Entropy loss (BCE) function [34] as in Equation (1):

(1) $$L_{Balanced-CE}\left(y,\hat{y}\right)=\left\{\beta \times y\log\left(\hat{y}\right)+\left(1-\beta \right)\times \left(1-y\right)\log\left(1-\hat{y}\right)\right\}$$

where $\hat{y}$ is the class SoftMax probability and y is the ground truth of the corresponding prediction. $\beta =1-\frac{y}{H\times W},$ and H×W presents the total of pixels in the image. Furthermore, β is used for adjusting the number of false negatives and false positives as follows: reducing the number of false negatives when β>1 or reducing the number of false positives when β<1.

The Balanced Cross-Entropy (BCE) loss function offers the following advantages:

- Unbalanced Data Processing: In binary classification problems, the BCE function addresses the issue of unbalanced data. It ensures that if the sample ratio between the two classes is unequal, the smaller sample will be considered more significant. This prevents the model from being biased towards the larger sample.
- Error balancing: The BCE function takes into account error levels in both classes. This causes the model to strive to minimize the mean error for both classes, as opposed to concentrating excessively on the minority class.
- Increased accuracy: By managing unbalanced data and equalizing errors, the BCE function can improve model accuracy in binary classification problems. It serves to balance class-based decisions and minimizes the impact of minority information.

Additionally, the BCE function can be applied to multilayer image segmentation issues. The BCE function is maximally efficient with a variety of datasets, particularly unbalanced datasets. Hence, path planning will function more effectively in a variety of internal environments.

**Figure 5:**
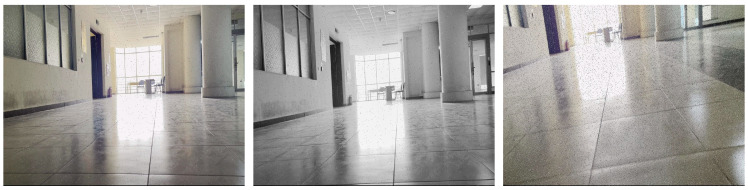
Pre-processed images of specific scene number 10, 100 and 110 in TaQuangBuu’s dataset.

To optimize the balanced cross-entropy defined in Equation (1), the Adam optimizer [35] was used. The model was trained with a learning rate of 0.001 and for 100 epochs. The dataset was pre-processed with Gaussian blur [36] (as defined in Equation (2)) and Gaussian noise [37] (as defined in Equation (3)) to ensure the quality of raw images before passing them through the proposed segmentation model shown in Figure 5. By using the aforementioned algorithms, the image quality will be altered, but it can create more generalized datasets, enhancing the segmentation model’s quality.

The Gaussian blur is an image filtering technique to calculate the transformation to each pixel in the image using a Gaussian function. In two dimensions, each dimension is shown below:

(2) $$G\left(x,y\right)=\frac{1}{2\pi \sigma^{2}}e^{-\frac{x^{2}+y^{2}}{2\sigma^{2}}}$$

where x: the horizontal distance from the origin; y: the vertical distance from the origin, σ: the standard deviation of the Gaussian distribution. It is essential to observe that the origin of these axes is centered (0, 0). Based on this formula, it generates a two-dimensional surface, the contours of which are concentric circles with a Gaussian distribution outward from the center.

In digital image processing, Gaussian noise will be reduced using a spatial filter. An undesirable consequence may be the blurring of fine-scaled image edges to smooth an image, meaning that one must make details corresponding to the blocked high frequencies. The probability density function p of a Gaussian random variable z is in Equation (3):

(3) $$p_{G}\left(z\right)=\frac{1}{\sigma \sqrt{2\pi }}e^{-\frac{{\left(z-\mu \right)}^{2}}{2\sigma^{2}}}$$

where z: the grey level, μ: the mean grey value, and σ: the standard deviation.

### 3.3. Quantization

**Figure 6:**
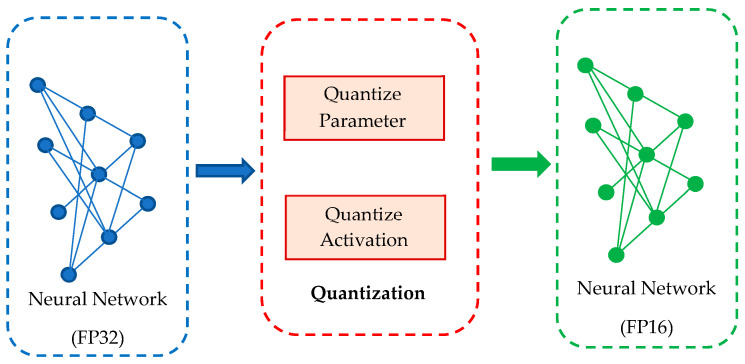
Proposed quantization process.

Quantization [38] is a technique that reduces the size and performance requirements of a machine-learning model by representing its parameters with reduced precision. Parameters such as the weight and bias of a neural network are frequently represented with high precision using floating-point data during the training phase of a machine-learning model. However, this requires a large amount of storage and computational resources, which can be a challenge when deploying the model on devices with limited resources, such as mobile devices or embedded microcontrollers. By quantizing the model, in other words, representing the model’s parameters as limited-precision integers or real-number data, the storage size can be reduced while the computational performance is increased. This can be achieved through quantization techniques such as weight quantization, activation quantization, or a combination of both. To minimize the model’s size, the model was converted from FP32 (32-bit floating-point precision) to FP16 (16-bit floating-point precision) during the training experiment shown in Figure 6. The quantization process typically includes two main steps as follows: Precision Calibration; Layer and Tensor Fusion.

- Precision Calibration: During training, FP32 (Floating Point 32) parameters and activations will be converted to FP16. Optimizing it will decrease stagnation and increase inference speed, but at the expense of a slight reduction in model accuracy. In real-time recognition, accuracy and inference speed must sometimes be compromised.
- Layer and Tensor Fusion: Layer and tensor merge are performed to optimize GPU memory and bandwidth by merging nodes vertically, horizontally, or both. Vertical merging involves joining successive kernel processes, while horizontal merging involves merging layers with the same layer size and input but differing weights into a single layer.

## 4. Experimental Results and Discussion

### 4.1. Quantitative Results

The proposed method was evaluated using three datasets, including CitySpaces’ dataset (5000 images) [31], KITTI’s dataset (400 images) [32], and Duckie’s dataset collected from Ducktown (1200 images) [33]. In addition, we collected a set of 1200 additional authentic images from the Ta Quang Buu library to enhance the dataset. There were sets of three specifications (Accuracy, Loss, and mIoU) considered for the segmentation model’s requirements. Many comparisons with previous methods were carried out. 

**Figure 7:**
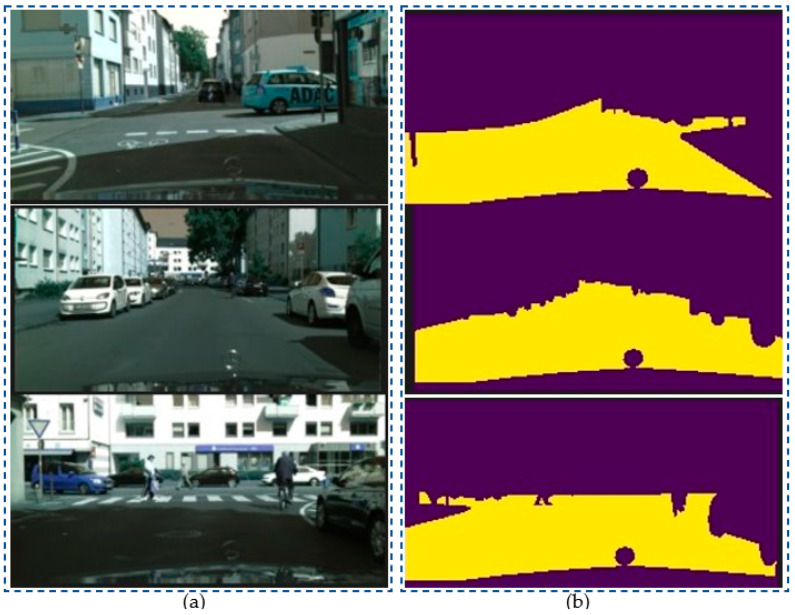
Segmentation results of specific scene number 1, 2 and 3 in Cityspaces’ dataset with (**a**) raw images and (**b**) segmented images.

Based on the input images of CitySpaces shown in Figure 4a, the segmented images in different conditions of environment had still been guaranteed with robust performance in Figure 7.

In addition, a number of baselines that have a similar encoder’s architecture were considered for evaluation, such as DSSPN [39], SqueezeNAS [40], and SaGe [41]. During the training process, the parameters and activation functions were represented in FP32. Consequently, switching to FP16 reduces latency and substantially reduces the model’s size. In fact, when converting to FP16, some weights will be reduced due to the smaller range of FP16 compared to FP32, resulting in a modest but insignificant decrease in accuracy. Table 1 illustrates that our segmentation model achieves the highest mIoU among the FCN models using the same Cityspaces’ dataset. Furthermore, our lightweight segmentation using reduced training parameters obtained higher validated mIoU, ranging from 2 to 10 percent. The accuracy and performance of the proposed segmentation model are ensured to construct the MR’s frontal view later.

**Figure 8:**
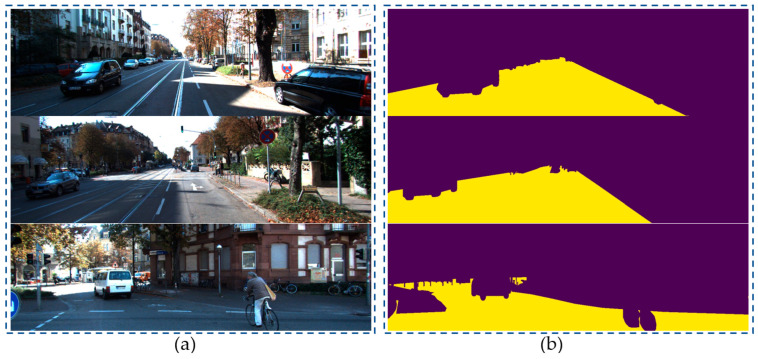
Segmentation results of specific scene number 1, 2 and 3 in KITTI-dataset with (**a**) raw images and (**b**) segmented images.

We conducted a comparison between our proposed model and existing segmentation models using the KITI dataset. Figure 4b shows the input images from the KITTI dataset, and Figure 8 demonstrates that the robust segmentation performance of the proposed method is still guaranteed. Moreover, the comparison with baselines such as SDNet [42], SFRSeg [43], and APMoE seg ROB [44] was also performed to prove the positive performance of the proposed methods. Table 2 presents the results of this comparison, showing that the proposed method outperforms the best-performing method, SDNet [39], by nearly 3% in terms of mIoU. Due to the expansion of our dataset with more challenging images and the extensive use of training data, our improved segmentation model using the quantization technique could acquire a more accurate representation of the environment and a faster training process. Thus, these achievements will strongly support constructing the MR’s frontal view. Finally, optimal path planning will be successfully designed. 

**Figure 9:**
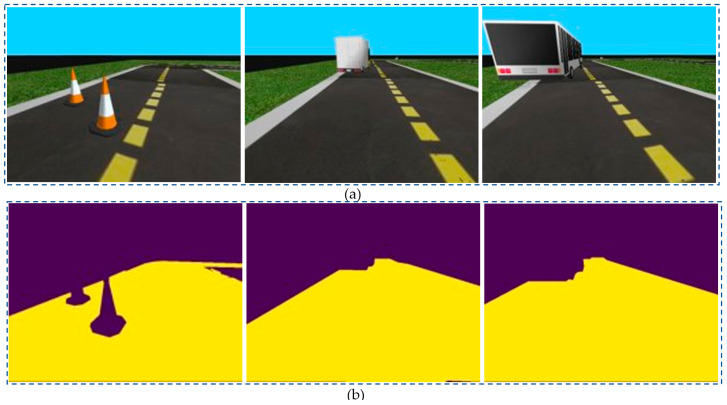
Segmentation results of specific scene number 7, 64 and 95 in Ducktown’s dataset with (**a**) raw images and (**b**) segmented images.

Furthermore, we self-gathered real-time images, finding it somewhat challenging to reflect upon. Next, 1200 images were collected from Ducktown’s dataset. The obtained experimental results have proven the feasibility of the proposed model’s application in realistic environments. In changing light conditions, our proposed model could correctly classify ground and non-ground regions, a circumstance that had typically been challenging for humans. This was because color-shifting training was undertaken, which enabled our network to operate effectively in low-light mode. Given the prevalence of corners and intersections in interior environments, the following examples were more intuitive for MRs. We executed the final performance evaluation on a background image containing numerous objects. Our network accurately anticipated the ground limit under challenging conditions, proving the robustness performance of segmented images, as depicted in Figure 9.

The proposed model’s performance has been consolidated and compared with the previous segmentation FCN-VGG 16 [7] on the same dataset, as shown in Table 3. In this case, we expanded the dataset with more challenging images. Then, to train the model more effectively, data augmentation was extensively used, resulting in a more accurate representation of the environment, followed by significant performance improvements.

**Figure 10:**
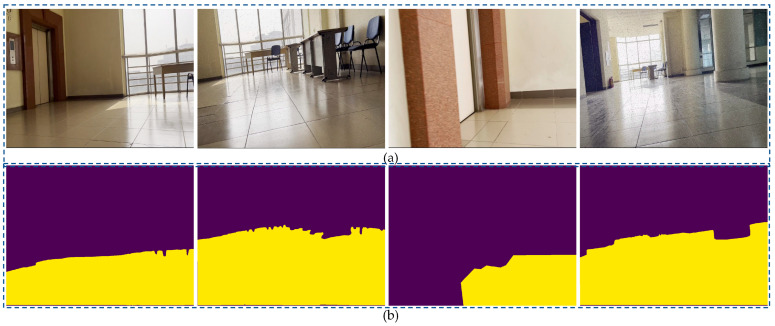
Segmentation results of specific scene number 690, 430, 730 and 380 in TaQuangBuu’s dataset consisting of obstacles, corners, and intersections with (**a**) raw images and (**b**) segmented images.

Finally, the authors continuously self-collected more than one thousand two hundred images from the TaQuangBuu library. The image input’s size was 960 × 1280. The final network performance evaluation was executed on a background image with numerous obstacles and intersections in Figure 10. 

**Figure 11:**
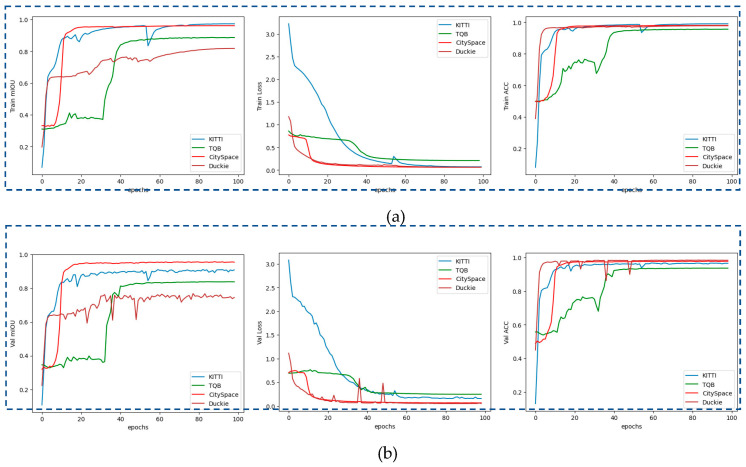
The diagrams of Accuracy, Loss, and mIoU in both of (**a**): training and (**b**): validation process.

**Figure 12:**
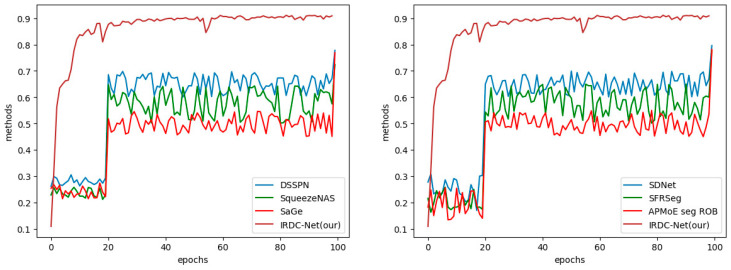
The comparison between our IRDC-Net and other methods.

Figure 11 illustrates the Accuracy, Loss, and mIoU diagrams in both the training and validation process. The diagrams depict the performance metrics when input images were taken from four datasets of CitySpaces, KITTI, Duck-town, and TaQuangBuu library, as shown in Figure 12.

**Figure 13:**
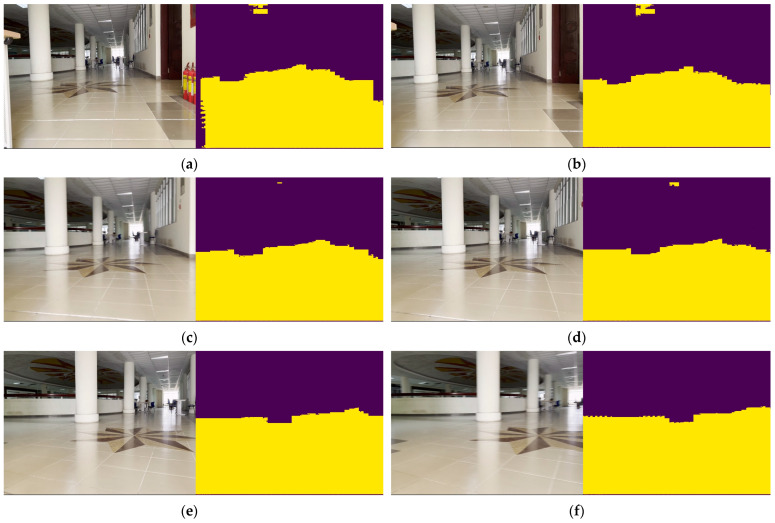

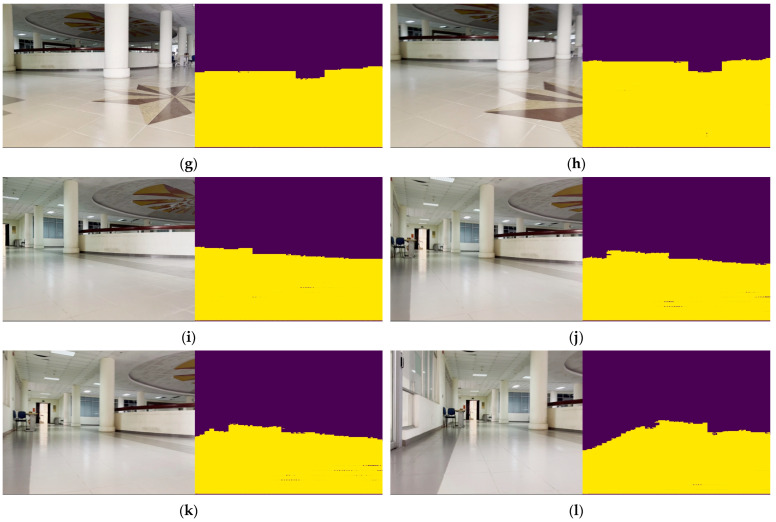
The segmented images captured by the mobile robot camera.

After undergoing color-shifting training, IRDC-Net’s performance is well remained when changing from low-light to dark scenarios. Indoor environments are full of corners and crossroads, making the following scenario more obvious to mobile robots. In Figure 13, the ground boundary in these challenging scenarios is reliably predicted by our network. Twelve snapshots from (a) to (l) of Figure 13 show illustrating the MR turning left to reach the goal point with the support of the local search algorithm.

### 4.2. Mobile Robot’s Frontal View

**Figure 14:**
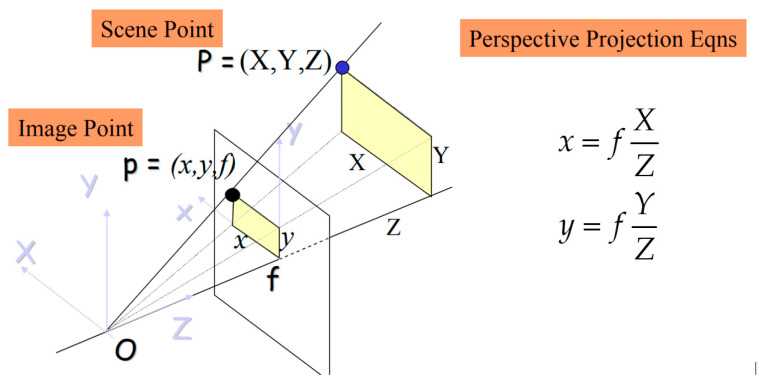
Basic perspective projection.

Firstly, based on the camera’s focal length, the point coordinates will be converted into the image plane. The relationship between the image plane and image coordination is shown in Figure 14.

Then, image coordinates can be rewritten in homogeneous coordination in Equation (4):

(4) $$\left(\begin{array}{ccc} x & y & 1 \end{array}\right)=\left(\begin{array}{ccc} f\frac{X_{C}}{Z_{C}} & f\frac{Y_{C}}{Z_{C}} & 1 \end{array}\right)$$

The transformation matrix projection from 3D image coordinates $\left(X_{C},Y_{C},Z_{C}\right)$ to 2D image plane (x,y):

(5) $$\left[\begin{array}{c} f_{X}\\ f_{Y}\\ Z \end{array}\right]=\left[\begin{array}{cccc} f & 0 & 0 & 0\\ 0 & f & 0 & 0\\ 0 & 0 & 1 & 0 \end{array}\right]\left[\begin{array}{c} X\\ Y\\ Z\\ 1 \end{array}\right]$$

**Figure 15:**
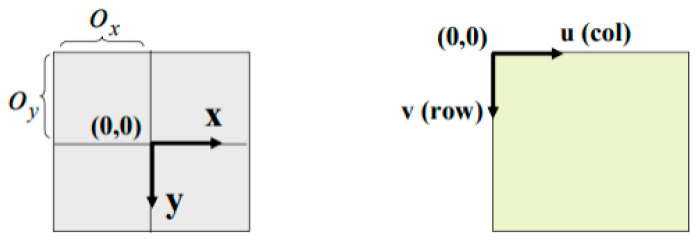
Image plane to pixel plane transformation.

Obtaining the result of Equation (5) yields the output of point coordinates in the image plane: P=(x,y). Then, the transformation from the image plane to the pixel plane will be carried out as follows in Figure 15.

The affine transformation is a translation in the 2D plane from Image Plane coordination (x,y) to Pixel Plane coordination (u,v) with $\left(O_{x},O_{y}\right)$: the image center (in Equation (6)):

(6) $$\{\begin{array}{c} x+O_{x}=f\frac{X_{C}}{Z_{C}}+O_{x}\\ y+O_{y}=f\frac{Y_{C}}{Z_{C}}+O_{y} \end{array}$$

Next, the pinhole camera model is derived with the help of homogenous coordinates and projective space. This model describes how to map a three-dimensional scene onto a two-dimensional picture with the help of the following Equation (7):

(7) $$\left[\begin{array}{c} u\\ v\\ \omega \end{array}\right]=\left[\begin{array}{cccc} f_{x} & 0 & 0 & 0\\ 0 & f_{y} & c_{y} & 0\\ 0 & 0 & 1 & 0 \end{array}\right]\left[\begin{array}{cccc} r_{11} & r_{13} & r_{13} & t_{1}\\ r_{21} & r_{22} & r_{23} & t_{2}\\ r_{31} & r_{32} & r_{33} & t_{3}\\ 0 & 0 & 0 & 1 \end{array}\right]\left[\begin{array}{c} X\\ Y\\ Z\\ 1 \end{array}\right]$$

The first transformation matrix presents an extrinsic camera matrix defining the camera’s position in the 3D environment. The second transformation matrix presents the intrinsic camera matrix converting the image plane (*x*,*y*) to the pixel plane (*u*,*v*).

**Figure 16:**
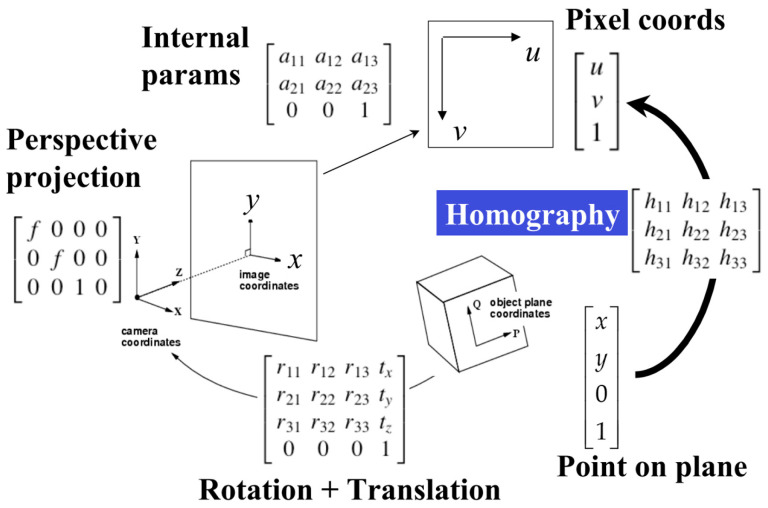
Homography transformation from the image plane to the actual ground region.

Moreover, in Figure 16, the authors use homography transformation to correct the perspective distortion, setting up the pixel plane to plan the MR’s path in order to present the relationship between world coordination (W: 4 × 1) and the image plane (*p*: 3 × 1). The expression of Equation (8) describes the transformation as follows:

(8) $$p=M_{\mathrm{int}}\times M_{\mathrm{ex}t}\times W$$

where *M*_int_ is the matrix of Intrinsic parameters (3 × 4) and $M_{\mathrm{ex}t}$ is the matrix of Extrinsic parameters (4 × 4). Using the mappings between 3D object points (points stated in the object frame) and the projected 2D image points (points in the object seen in the image), we can determine the camera’s orientation. As for Equation (5), the treated image as a ground surface Z=0, if the camera poses [45], is fixed to the MR (rotating only on *Z*-axis) shown in Equation (9).

(9) $$\left[\begin{array}{c} u\\ v\\ 1 \end{array}\right]∼\left[\begin{array}{cccc} f & 0 & 0 & 0\\ 0 & f & 0 & 0\\ 0 & 0 & 1 & 0 \end{array}\right]\times \left[\begin{array}{cccc} r_{11} & r_{12} & r_{13} & t_{x}\\ r_{21} & r_{22} & r_{23} & t_{y}\\ r_{31} & r_{32} & r_{33} & t_{z}\\ 0 & 0 & 0 & 1 \end{array}\right]\times \left[\begin{array}{c} x\\ y\\ 0\\ 1 \end{array}\right]$$

For the planar surface Z=0, the expression (9) can be rewritten as the following (Equation (10)):

(10) $$\left[\begin{array}{c} u\\ v\\ 1 \end{array}\right]∼\left[\begin{array}{ccc} h_{11} & h_{12} & h_{13}\\ h_{21} & h_{22} & h_{23}\\ h_{31} & h_{32} & h_{33} \end{array}\right]\times \left[\begin{array}{c} x\\ y\\ 1 \end{array}\right]=H\times \left[\begin{array}{c} x\\ y\\ 1 \end{array}\right]$$

Based on Equations (4)–(10), the mobile robot’s frontal view is designed to plan the MR’s path. All steps are summarized in Figure 16.

Finally, applying homography parameters estimation H with the relation between perspective plane one (x,y) and perspective plane two (x′,y′) [7,10]:

(11) $$\left\{ \begin{array}{l} x=\frac{h_{11}\times x\prime +h_{12}\times y+h_{13}}{h_{31}\times x\prime +h_{32}\times y+1}\\ y=\frac{h_{21}\times x\prime +h_{22}\times y+h_{23}}{h_{31}\times x\prime +h_{32}\times y+1} \end{array}\right.$$

**Figure 17:**
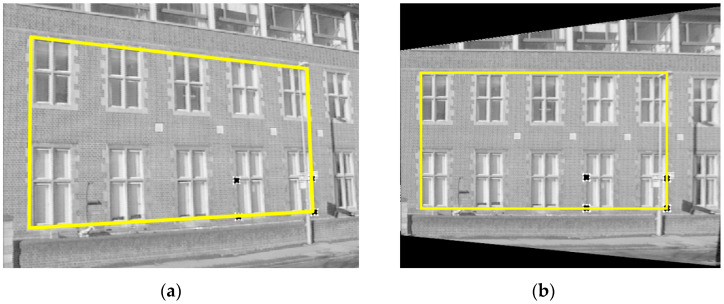
Homography transformation for four-point correspondences suffice for the planar surface with (**a**) image plane and (**b**) pixel plane in MR’s frontal view [46].

**Figure 18:**
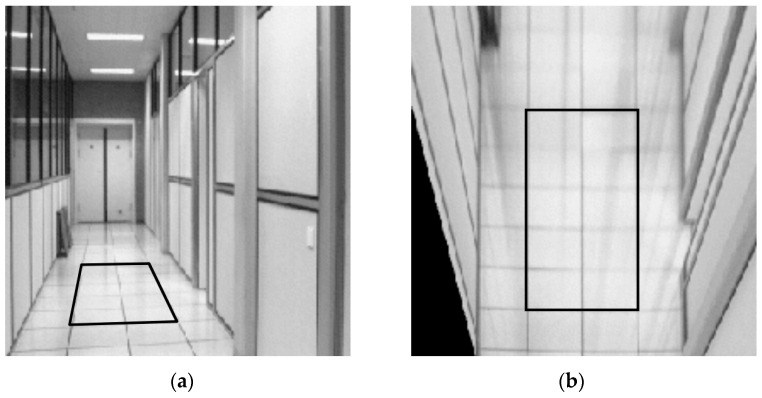
The homography transformation for bird’s eye view of MR with (**a**) image plane and (**b**) pixel plane in bird’s eye view [46].

**Figure 19:**
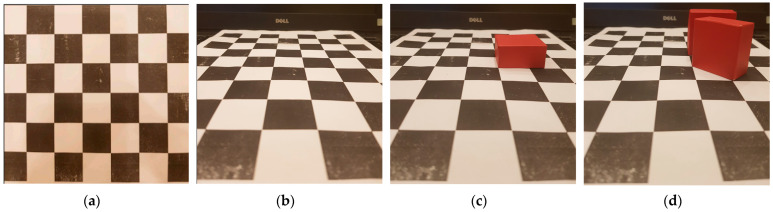
The frontal view of mobile robot with (**a**) checkerboard, (**b**) frontal view, (**c**) one obstacle and (**d**) two obstacles.

Thus, using two sets of four known points (x,y) and (x′,y′) to calculate the H matrix, any four points of the pixel plane in the MR’s frontal view will be wholly obtained in Figure 17. Furthermore, the homography transformation for the bird’s eye view of MR is shown in Figure 18. Using the checkerboard in Figure 19a, the authors perform the perspective correction of MR’s frontal views (see Figure 19b). Based on the segmented image, the allowance moving areas will be proportional to the ground coordinate. The positions of MR and obstacles are entirely determined to design the path planning in Figure 19c,d.

### 4.3. Practical Results

**Figure 20:**
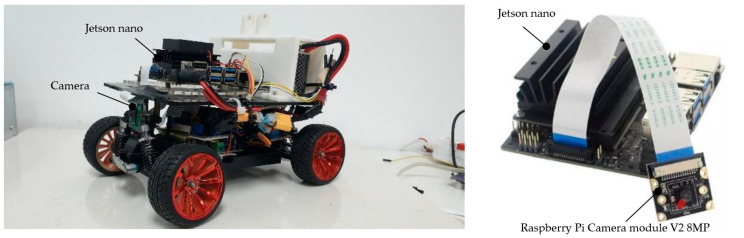
Mobile robot with Jetson nano and monocular camera.

In comparison to a previous study [10], the same optimal MR’s strategy [10] was used. However, in this study, the proposed FCN-MobilenetV2 model was utilized to obtain segmented images, facilitating constructing the available area for movement, as depicted in Figure 20. 

**Figure 21:**
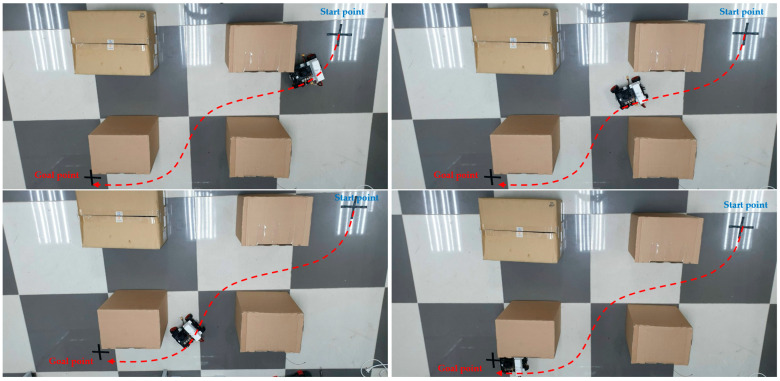
MR tracks the path based on the proposed semantic segmentation model.

Furthermore, a dedicated local search algorithm was designed to increase the safety of obstacle avoidance when the MR successfully tracks the global path, as shown in Figure 21. After analyzing the new results compared with those obtained in [7], we could draw the conclusion that semantic segmentation is necessary when constructing the ground’s frontal perspective. This enables the planning of the most efficient path for MR. The practical experiments conducted in this study focused on enhancing methods for recognizing collision-free zones in local search areas based on the given global path.

**Figure 22:**
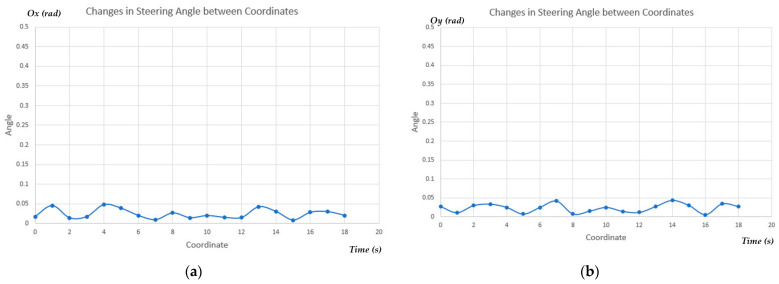
The influence of changes in steering between coordinates with (**a**) rotation around *x*-axis and (**b**) rotation around *y*-axis.

Since the camera pose was fixed to the MR, a smooth trajectory would affect the performance of the proposed semantic segmentation. In other words, our proposed model would ensure better results compared to previous FCN-VGG 16 [7] with model parameters in Table 3. When being tested on multiple datasets, our proposed model exhibited a remarkable quality enhancement, as shown in Figure 12. Table 4 presented the relationship between changing the steering angle and the accuracy of proposed model, considering the fixed camera pose. It demonstrated that as the steering angle increases, the accuracy of the model decreases significantly. Therefore, our improved semantic segmentation would ensure the smoothness of MR’s trajectory and maintain the minimum changes in the steering angle, as depicted in Figure 22. Thus, a smooth trajectory with low steering angle changing would improve the performance of our lightweight semantic segmentation FCN-MobilenetV2 in MR’s movement. 

## 5. Conclusions

This paper proposes a real-time solution to extract corridor scenes from a single image supporting mobile robot navigation. Specifically, a lightweight semantic segmentation model that integrates a quantization technique is introduced to improve the segmentation accuracy while achieving a low computational cost. The evaluation results are compared with recent methods to demonstrate the feasibility of the proposed method. Moreover, our proposed lightweight semantic segmentation FCN-MobilenetV2 can be significantly better in terms of precision and computation time, compared to the previous semantic segmentation FCN-VGG-16. The practical result shows the successful tracking of the mobile robot’s path with a lower 0.05 rad steering angle change. Indeed, our proposed segmentation model is trained and updated from binary classes to multi-classed to identify a wide variety of internal barriers accurately. Therefore, in a real situation, path planning will work better in a variety of indoor settings. In addition, the segmented results will support the local search algorithm in mobile robot path planning. Finally, the safety and avoidance abilities of MR are enhanced against static and dynamic obstacles in unknown environments.

## Figures and Tables

**Table 1:** The comparison among our proposed segmentation model with other models using Cityspaces’ dataset.

Model	Validated mIoU
DSSPN [39]	77.8%
SqueezeNAS [40]	72.4%
SaGe [41]	76.9%
IRDC-Net: Lightweight Segmentation	78.1%

**Table 2:** The comparison among our proposed segmentation model with other models using KITTI- dataset.

Model	Validated mIoU
SDNet [42]	79.62%
SFRSeg [43]	77.91%
APMoE seg ROB [44]	78.11%
IRDC-Net: Lightweight Segmentation	81.11%

**Table 3:** The comparison of proposed segmentation model with FCN-VGG 16 [7] using Ducktown’s dataset.

Model	Accuracy	Validated mIoU
Binary Segmentation FCN-VGG 16 [7]	97.1%	71.8%
IRDC-Net: Lightweight Segmentation	98.3%	74.2%

**Table 4:** The comparison of the proposed segmentation model with previous segmentation models [7].

Frontal View of Mobile Robot	Steering Angle Changing (Rad)	Accuracy (%)
When rotating around X axis	0.00	100
	0.01	96.5
	0.02	93.2
	0.03	91.4
	0.04	88.6
	0.05	85.3
	0.07	82.5
	0.09	81.1
When rotating around Y axis	0.00	100
	0.03	99
	0.05	94.6
	0.07	92.4
	0.09	90.2
	0.1	87.8
	0.12	83.2
	0.15	80.1

## Data Availability

Taquangbuu library’s dataset is supplied as the following link: https://github.com/dangthaiviet/TaQuangBuu.Dataset (accessed on 31 July 2023).

## References

[1] Murat L., Ertugrul C., Faruk U., Ibrahim U., Salih I.A. (2018). Initial Results of Testing a Multilayer Laser Scanner in a Collision Avoidance System for Light Rail Vehicles. Appl. Sci..

[2] Abukhalil T., Alksasbeh M., Alqaralleh B., Abukaraki A. (2020). Robot navigation system using laser and monocular camera. J. Theor. Appl. Inf. Technol..

[3] Wang W.C., Ng C.Y., Chen R. (2021). Vision-Aided Path Planning Using Low-Cost Gene Encoding for a Mobile Robot. Intell. Automat. Soft Comput..

[4] Maulana I., Rasdina A., Priramadhi R.A. (2018). Lidar applications for Mapping and Robot Navigation on Closed Environment. J. Meas. Electron. Commun. Syst..

[5] Damodaran D., Mozaffari S., Alirezaee S., Ahamed M.J. (2023). Experimental Analysis of the Behavior of Mirror-like Objects in LiDAR-Based Robot Navigation. Appl. Sci..

[6] Al-Mallah M., Ali M., Al-Khawaldeh M. (2022). Obstacles Avoidance for Mobile Robot Using Type-2 Fuzzy Logic Controller. Robotics.

[7] Dang T.V., Bui N.T. (2023). Multi-Scale Fully Convolutional Network-Based Semantic Segmentation for Mobile Robot Navigation. Electronics.

[8] Zhao C.Q., Sun Q.Y., Zhang C.Z., Tang Y., Qian F. (2020). Monocular depth estimation based on deep learning: An overview. Sci. China Technol. Sci..

[9] Dong Q. (2022). Path Planning Algorithm Based on Visual Image Feature Extraction for Mobile Robots. Mob. Inf. Syst..

[10] Dang T.V., Bui N.T. (2023). Obstacle Avoidance Strategy for Mobile Robot Based on Monocular Camera. Electronics.

[11] Pan X., Gao L., Marinoni A., Zhang B., Yang F., Gamba P. (2018). Semantic Labeling of High Resolution Aerial Imagery and LiDAR Data with Fine Segmentation Network. Remote Sens..

[12] Peng C., Li Y., Jiao L., Chen Y., Shang R. (2019). Densely Based Multi-Scale and Multi-Modal Fully Convolutional Networks for High-Resolution Remote-Sensing Image Semantic Segmentation. IEEE Trans. Sel. Top. Appl. Earth Obs. Remote Sens..

[13] Wang Y., Sun Z., Zhao W. (2021). Encoder- and Decoder-Based Networks Using Multi-scale Feature Fusion and Nonlocal Block for Remote Sensing Image Semantic Segmentation. IEEE Geosci. Remote Sens. Lett..

[14] Pastorino M., Moser G., Serpico S.B., Zerubia J. (2022). Semantic Segmentation of Remote-Sensing Images through Fully Convolutional Neural Networks and Hierarchical Probabilistic Graphical Models. IEEE Geosci. Remote Sens..

[15] Lyu C., Hu G., Wang D. (2020). HRED-Net: High-Resolution Encoder-Decoder Network for Fine-Grained Image Segmentation. IEEE Access.

[16] Rusli L., Nurhalim B., Rusyadi R. (2021). Vision-based vanishing point detection of autonomous navigation of mobile robot for outdoor applications. J. Mechatron. Elect. Power Veh. Technol..

[17] Minaee S., Boykov Y.Y., Porikli F., Plaza A.J., Kehtarnavaz N., Terzopoulos D. (2022). Image Segmentation Using Deep Learning: A Survey. IEEE Trans. Pattern Anal. Mach. Intell..

[18] Shelhamer V., Long J., Darrell T. (2016). Fully Convolutional Networks for Semantic Segmentation. IEEE Trans. Pattern Anal. Mach. Intell..

[19] Wang C., Zhao Z., Ren Q., Xu Y., Yu Y. (2019). Dense U-Net based on patch-based learning for retinal vessel segmentation. Entropy.

[20] Wang W., Yu K., Hugonot J., Fua P., Salzmann M. Recurrent U-Net for resource-constrained segmentation. Proceedings of the IEEE/CVF International Conference on Computer Vision (ICCV).

[21] Agus E.M., Bagas Y.S., Yuda M., Hanung A.N., Zaidah I. (2022). Convolutional Neural Network featuring VGG-16 Model for Glioma Classification. Int. J. Inform. Vis..

[22] Alfred Daniel J., Chandru Vignesh C., Muthu B.A., Senthil Kumar R., Sivaparthipan C.B., Marin C.E.M. (2023). Fully convolutional neural networks for LIDAR-camera fusion for pedestrian detection in autonomous vehicle. Multimed. Tools Appl..

[23] Cruz R., Silva D.T., Goncalves T., Carneiro D., Cardoso J.S. (2023). Two-Stage Framework for Faster Semantic Segmentation. Sensors.

[24] Kong X., Xia S., Liu N., Wei M. (2023). GADA-SegNet: Gated attentive domain adaptation network for semantic segmentation of LiDAR point clouds. Vis. Comput..

[25] Badrinarayanan V., Alex K., Roberto C. (2017). Segnet: A deep convolutional encoder-decoder architecture for image segmentation. IEEE Trans. Pattern Anal. Mach. Intell..

[26] Paszke A., Chaurasia A., Kim S., Culurciello E. (2016). Enet: A deep neural network architecture for real-time semantic segmentation. arXiv.

[27] Wang Y. (2022). Remote sensing image semantic segmentation network based on ENet. J. Eng..

[28] Qin Y., Tang Q., Xin J., Yang C., Zhang Z., Yang X. (2023). A Rapid Identification Technique of Moving Loads Based on MobileNetV2 and Transfer Learning. Buildings.

[29] Wang P., Luo F., Wang L., Li C., Niu Q., Li H. (2022). S-ResNet: An improved ResNet neural model capable of the identification of small insects. Front. Plant Sci..

[30] Gao L., Huang Y., Zhang X., Liu Q., Chen Z. (2022). Prediction of Prospecting Target Based on ResNet Convolutional Neural Network. Appl. Sci..

[31] Cordts M., Omran M., Ramos S., Rehfeld T., Enzweiler M., Benenson R., Franke U., Roth S., Schiele B. The Cityscapes Dataset for Semantic Urban Scene Understanding. Proceedings of the IEEE Conference on Computer Vision and Pattern Recognition (CVPR).

[32] Hassan A., Siva M., Lars M., Andreas G., Carsten R. (2018). Augmented Reality Meets Computer Vision: Efficient Data Generation for Urban Driving Scenes. Int. J. Comput. Vis. (IJCV).

[33] Kirill K., Konstantin C., Anton F., Artyom F. (2021). Autonomous Wheels And Camera Calibration In Duckietown Project. Procedia Comput. Sci..

[34] Quentin J., Liu X., Murata T. (2022). Balanced softmax cross-entropy for incremental learning with and without memory. Comput. Vis. Image Underst..

[35] Liu M., Yao D., Liu Z., Guo J., Chen J. (2023). An Improved Adam Optimization Algorithm Combining Adaptive Coefficients and Composite Gradients Based on Randomized Block Coordinate Descent. Comput. Intell. Neurosci..

[36] Kostková J., Flusser J., Lébl M., Pedone M. (2020). Handling Gaussian Blur without Deconvolution. Pattern Recognit..

[37] Aghajarian M., McInroy J.E., Muknahallipatna S. (2020). Deep learning algorithm for Gaussian noise removal from images. J. Electron. Imag..

[38] Tsubota K., Aizawa K. (2023). Comprehensive Comparisons of Uniform Quantization in Deep Image Compression. IEEE Access.

[39] Liang X., Hongfei Z., Eric X. Dynamic-structured semantic propagation network. Proceedings of the IEEE Conference on Computer Vision and Pattern Recognition.

[40] Shaw A., Hunter D., Landola F., Sidhu S. Squeezenas: Fast neural architecture search for faster semantic segmentation. Proceedings of the IEEE/CVF International Conference on Computer Vision Workshops.

[41] Tian Y., Xie L., Zhang X., Fang J., Xu H., Huang W., Jiao J., Tian Q., Ye Q. (2021). Semantic-Aware Generation for Self-Supervised Visual Representation Learning. arXiv.

[42] Ochs M., Kretz A., Mester R. SDNet: Semantic Guided Depth Estimation Network. Proceedings of the 41st DAGM German Conference, DAGM GCPR 2019.

[43] Singha T., Pham D., Krishna A. (2023). A real-time semantic segmentation model using iteratively shared features in multiple sub-encoders. Pattern Recognit..

[44] Kong S., Fowlkes C. (2018). Pixel-wise Attentional Gating for Parsimonious Pixel Labeling. arXiv.

[45] Marchand E., Uchiyama H., Spindler F. (2016). Pose Estimation for Augmented Reality: A Hands-On Survey. IEEE Trans. Vis. Comput. Graph..

[46] Hartley R., Xisserman A. (2000). Multiple View Geometry in Computer Vision.

